# Efficacy of the small step program in a randomised controlled trial for infants below age 12 months with clinical signs of CP; a study protocol

**DOI:** 10.1186/s12887-016-0711-x

**Published:** 2016-11-03

**Authors:** Ann-Christin Eliasson, Linda Holmström, Päivikki Aarne, Cecilia Nakeva von Mentzer, Ann-Louise Weiland, Lena Sjöstrand, Hans Forssberg, Kristina Tedroff, Kristina Löwing

**Affiliations:** 1 Department of Women’s and Children’s Health, Karolinska Institutet, 171 76 Stockholm, Sweden; 2 Division of Speech and Language Pathology, Department of CLINTEC, Karolinska Institutet, Stockholm, Sweden; 3 Department of Speech and Language Pathology, Karolinska University Hospital, Stockholm, Sweden; 4 Unit for Speech Language Pathology, Department of Neuroscience, Uppsala University, Uppsala, Sweden

**Keywords:** Cerebral palsy, Infant, Early intervention, Fine motor function, Gross motor function, Communication

## Abstract

**Background:**

Children with cerebral palsy (CP) have life-long motor disorders, and they are typically subjected to extensive treatment throughout their childhood. Despite this, there is a lack of evidence supporting the effectiveness of early interventions aiming at improving motor function, activity, and participation in daily life. The study will evaluate the effectiveness of the newly developed Small Step Program, which is introduced to children at risk of developing CP during their first year of life. The intervention is based upon theories of early learning-induced brain plasticity and comprises important components of evidence-based intervention approaches used with older children with CP.

**Method and design:**

A two-group randomised control trial will be conducted. Invited infants at risk of developing CP due to a neonatal event affecting the brain will be randomised to either the Small Step Program or to usual care. They will be recruited from Astrid Lindgren Children’s Hospital at regular check-up and included at age 3–8 months. The Small Step Program was designed to provide individualized, goal directed, and intensive intervention focusing on hand use, mobility, and communication in the child’s own home environment and carried out by their parents who have been trained and coached by therapists. The primary endpoint will be approximately 35 weeks after the start of the intervention, and the secondary endpoint will be at 2 years of age. The primary outcome measure will be the Peabody Developmental Motor Scale (second edition). Secondary assessments will measure and describe the children’s general and specific development and brain pathology. In addition, the parents’ perspective of the program will be evaluated. General linear models will be used to compare outcomes between groups.

**Discussion:**

This paper presents the background and rationale for developing the Small-Step Program and the design and protocol of a randomized controlled trial. The aim of the Small Step Program is to influence development by enabling children to function on a higher level than if not treated by the program and to evaluate whether the program will affect parent’s ability to cope with stress and anxiety related to having a child at risk of developing CP.

**Trial registration:**

ClinicalTrials.gov Identifier NCT02166801. Registered June 12, 2014.

## Background

Cerebral Palsy (CP) is a result of a spectrum of early acquired disturbance to the developing brain. The brain abnormalities or insults that cause CP can appear at different time points during pregnancy and in the neonatal period [1]. To date, there are no biological or pharmacological treatments. However, neurobiological research suggests that the effectiveness of the neural networks and pathways can be strengthened by early interventions. Despite the rather large population of individuals with CP world-wide, there is still a lack of evidence-based early intervention programs that make it possible to utilize the therapeutic window that the rapid brain development and adaptability of the central nervous system during the first year of life represents [2, 3]. This paper describes the rationale, contents, and structure of the *Small Step Program*, an early intervention program for children at risk of developing CP. The Small Step Program was designed to provide individualized, goal directed, and intensive intervention within three distinct foci (hand use, mobility, and communication) in the child’s own home environment and carried out by their parents who have been educated and coached by therapists.

A plausible reason for the lack of effective early intervention programs for children at risk of developing CP is the common “wait and see” approach due to the uncertainty of early diagnostic criteria [3]. There is a wide range of factors, very often in combination, that can be the cause of the early insult to the developing brain that will result in the diagnosis of CP [4–6]. Highly individual developmental trajectories of postural control, motor function, and muscle tone that vary considerably during the first years are frequently seen in children at risk of developing CP. In addition, risk factors and signs of CP might be visible at a very early age but might not necessarily lead to CP. A child with delayed development might recover and atypical signs might disappear, and the opposite can occur in which a child develops CP without obvious early signs. Thus, there is the dilemma in that the potential effect of early intervention might be lost if we wait with intervention until the CP diagnosis has been confirmed. Although the ability to predict the risk of developing CP at an early age has increased when using a combination of general movement assessments, neurological examinations, and brain imaging techniques [7, 8]. Different brain structures are more susceptible to injury during certain time periods during pregnancy. An early insult during the first trimester can lead to a maldevelopment of the brain, while the white matter, particularly in areas close to the ventricles, is more vulnerable to injury in the late second to early third trimester. An insult close to term causes predominantly cortical and subcortical grey matter lesions (e.g. injuries involving the thalamus and basal ganglia) [9, 10]. The timing of the brain disturbance, in addition to its size and location, has been reported to be closely related to the severity of motor impairments [9, 11]. The effects of specific disturbance characteristics on motor development are understood to some extent; however, very little is known about the relationship between the timing and extent of the lesion and the effects of early interventions.

### Evidence for the efficacy of early intervention

Early intervention, carried out during the first year in life when the brain is under rapid development and most plastic, is assumed to be more effective than intervention later during development or in adulthood. This assumption is based on the knowledge that neural networks and pathways that remain intact after brain injury can be strengthened through learning-induced plasticity. Recent animal studies have suggested that there is a critical period of motor system plasticity and that activity-dependent reorganisation of the corticospinal motor-projection pattern to the hand occurs before 1 year of age [12, 13].

The potential of early interventions is well founded in experimental research, while clinical studies on physiotherapy for infants with CP, or children with high risk for CP, have not been able to show positive changes on motor development that exceed the expected developmental trajectory [14–17]. It seems that the concept of enriched environments based on great developmental effects in animal research can be transformed to children [18]. A recent pilot study using enriched environments to elicit self-initiated motor behaviours; including parent training and task analysis for frequent practice – has shown promising results [19]. Another recent early intervention study – COPing with and CAring for Infants with Special Needs (COPCA) – although vague results, is also of interest. COPCA is built of modern theories of brain development and uses the neuronal group selection theories framework in a family-centred approach [20]. There is some evidence indicating that more specific training might lead to a more favourable outcome when compared to general training [15]. For example, training approaches targeting other areas than overall motor development, such as cognition, seem to have positive results, at least in a short-term perspective, in preterm children [16, 21]. Early intervention directed towards communication is also known to have a positive influence on cognitive and pre-linguistic development and to result in more advanced communication in preterm children in a short term perspective [22]. When it comes to interventions targeting hand function, there is no specific intervention program developed for children with a high risk of developing CP. However, for children with unilateral CP, there are studies indicating that Constraint-Induced Movement Therapy (CIMT) can be adapted and used for infants and that this can increase the odds of having a higher functional level when the training is introduced within the first year of life [23]. From a family perspective, it is also of interest to highlight that early interventions have been shown to facilitate bonding and to reduce parental stress [8, 24]. These positive changes following intervention within the areas of cognition, communication, and attachment provide an interesting and promising contrast to the absence of improvements in overall motor development following intervention for infants. This is especially the case because there are high levels of evidence for several different treatment approaches aiming to improve motor function in older children [17]. Thus, there is a great need to develop new methods to improve models for early interventions in younger children and infants at high risk of developing CP.

### Theoretical background for the small step program *–* the child’s perspective

The Small Step Program is an individualized, goal directed, and intensive intervention that focuses on hand use, mobility, and communication. The program is intended to be carried out in the child’s home environment and be conducted by their parents who are trained and coached by therapists. The program is designed be used from about 4 months of age and seeks to take advantage of the theoretically plastic period of brain development at this age. The program is designed to include and combine components that are known to be of importance in evidence-based intervention approaches for older children. The challenge in the Small Step Program is to adapt this knowledge and apply it within an intervention for very young children.

The goal-directed approach has been successfully used in older children [25, 26]. However, for families in a very vulnerable time period of life, with a young infant with high risk of developing CP, it is not easy to define goals in either the short or long-term perspective. In a goal-setting approach, the person’s own individual wishes are a strong ingredient, and it is suggested that the goals should be functional and meaningful for daily life [27]. Such a goal-centred approach is taken in the Small Step Program, and collaboration between the parents and therapists will help the family to determine and focus on what their infant will likely be able to learn as a next step within the different foci and to define goals that are closely related to the child’s ability and cognitive level.

Within the Small Step Program, great emphasis is put on children’s self-initiated actions, which are stimulated by meaningful and motivating activities and toys [28, 29]. The stimuli should be clear and understandable for the child and should be related to the child’s developmental stage. It is assumed that development is driven by children’s unique characteristics and capacity to explore a situation through which they discover new and more advanced activities. Therefore, the training should be conducted at a level that is challenging, but not too demanding, i.e. the training should fall within the zone of proximal development, ZPD [30]. The scaffolding and shared attentional focus provided by the ZPD aims to ensure that the child’s interest is maintained for as long as possible, which will promote the internalisation of independent developmental achievements. This concept is commonly applied within motor learning programs. The challenge here lies in helping parents understand how self-initiated actions can be promoted for infants with a very low ability level.

Another important principle of the program is the concept of repetition and extensive practice. It is well established that a task has to be well learned if it is to be performed effectively. Thus, it must be possible even for infants to experience that a particular task can be performed with ease and success, such as grasping a toy or keeping control of their gaze. When Adolph and colleagues studied factors that could explain the rate of improvement when toddlers with typical development learn to walk, the only significant factor found was the amount of experience and practice that children had [31]. In the Small Step Program, we commonly use the word training, and in this sense it is synonymous to practice. It has to be emphasized that training can only be performed as long as the infant finds it enjoyable because infants cannot be forced to perform self-initiated actions. One of the overarching goals with the Small Step Program is thus to create a positive reflective atmosphere that promotes self-initiated actions and communication and communication in the child’s home environment. Maximizing the frequency of self-initiated motor activity in the child’s everyday environment has been suggested to be the most crucial factor supporting motor learning [32].

The rationale for dividing the intervention program into the three focus areas of hand use, mobility, and communication is to facilitate the learning process within each area. Typically, a child learns a lot within each area during the first year of life, and each focus area includes a huge amount of competence for parents as the training providers to capture. There is a limited amount of time during the day when a small child can attend to practice because caring and sleeping usually predominate their days. By introducing specific foci, we intend to help the family to optimize the training and to use their time as efficiently as possible. The *hand-use* focus will be used to help the child explore their external world through object manipulation, and this creates an important link to the child’s cognitive development. Knowledge about fine motor development and object exploration will guide the introduction of new activities. Voluntary action starts to emerge at birth, and von Hofsten and Lindhagen have demonstrated that reaching and grasping actions can be detected even in new-borns, though it takes some months before grasping actions are obvious and frequent [33]. In the first year of life, infants gradually gain remarkable control over their hands, and they begin exploring and manipulating objects with increasing skill [34, 35]. The *mobility* focus will be used to reach new gross motor ability goals with the overall aim that children should be able to move about and explore their external world on their own. This includes activities like maintaining and changing body position, sitting and standing, and any other form of mobility. The principles for reaching goals will be based on matching the child’s present capacity and environment with the target actions [36]. As a consequence, motor learning will not be expected to occur in the same order as in children with typical development. However, we do expect accelerated motor development to occur because there is a rapid pace during the first year of life during typical development. The *communication* focus will be used to enhance sensitive parental communication strategies that support child-centred interactions and child initiatives. The infants’ early communication skills will cover a wide range of behaviours such as crying, body movements, gestures, and directed gaze. These communication signals are the foundation for later linguistic skills and cognitive development. An important landmark to develop is shared focus or joint attention, i.e. the mutual attention between the infant and the parent. A particularly important form of joint attention is triadic engagement in which the infant can alternate his/her gaze between the parent and an outside object or event. Joint attention forms both a cognitive as well as a socio-emotional foundation for the child’s learning during activities and for their inter-personal engagement [37–39]. Raising awareness of the child’s communicative attempts and learning to respond and maintain communication will guide the training.

The intervention within the different foci will be further described in the methods section.

### Coaching, education, and collaboration with parents

Coaching of parents plays a prominent role in the Small Step Program. The aim is to provide a solution-focused approach that helps families to achieve goals that are unique and meaningful to them. There is at present no universal definition of coaching as a concept – definitions range from a relationship-directed to intervener-directed process – and there is no clear distinction between parent training and coaching [40]. Graham and colleagues’ definition of coaching contains emotional support, information exchange, and a structured process that includes elements such as goal setting, exploring options, planning actions, carrying out plans, checking performance, and generalizing. Furthermore, it is shown that the coach facilitates goal performance and supports parents in identifying ways to promote successful performance [41]. When coaching was used as a major strategy in a recent early intervention program (COPCA), it was defined as the promotion of creative exploration of the competencies of the family with the aim to encourage self-made decisions in everyday activities [42]. Further aspects that promote learning are presented by Turner and Paris, who emphasize the importance of motivation. They describe a model that supports learning called the “Six-C model” for choice, control, challenge, collaboration, constructive meaning, and consequence [43]. Although coaching is well established from a theoretical perspective, its effectiveness in practice has not been fully evaluated. A recent review summarizing the evidence for coaching’s effectiveness found only a few high-quality studies [44]. The most clear high-quality evidence was for improved educational outcomes for children at risk of developmental delay, but there was also some moderate evidence for improving motor outcome in children with CP if a motor learning approach is used. In the Small Step Program, we will take a broad approach to coaching and parent training. For the therapists coaching the parents to conduct the Small Steps training program, motivational interviews [45] and solution-focused coaching [32] are important tools.

## Methods and design

This paper describes the methodology of a randomised controlled trial comparing the effect of the Small Step Program with treatment as usual for children younger than 1 year of age who are at risk of developing CP or other neurodevelopmental disorders. The outcomes of this trial will be evaluated after 14 weeks of intervention, at the end of the study period (approximately 35 weeks after the start of the intervention) and when the children are 24 months corrected age.

### Primary and secondary objectives

The primary objective of this trial is to evaluate the effects of the newly developed Small Step Program on general development in children at risk for developing CP or other neurodevelopmental disorders. The main hypothesis of the study is that the Small Step Program is more effective than treatment as usual when children are evaluated at the age of 2 years. The rapid brain development and plasticity during the first year of life is utilized as a possible therapeutic window for intensive training. The design of the program, with three different foci during separate time periods, was designed in relation to the second hypothesis. We assume that children will have a more rapid development within the specific focus of each time period compared to the development rate within the other two foci that are not being trained at that particular time: i.e. you learn what you practice. The third hypothesis is that children’s ability to develop will be influenced by the specific characteristics of any underlying brain pathology.

The secondary objective is to evaluate the effect of the Small Step Program on parents’ ability to cope with the stress and anxiety related to the traumatic experience of parenting a child at risk of developing CP. The hypothesis is that parents in the Small Step Program will be less stressed and can better cope with their child’s situation than parents of children receiving usual care. Thus, the tools provided within the Small Step Program like coaching, supervision, education, and feedback on how to stimulate task performance and communication will make parents more able to cope with the child’s delayed development.

**Fig. 1:**
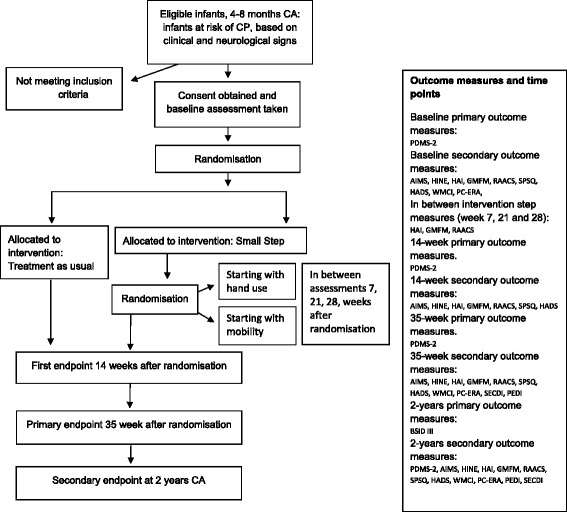
Consort flow chart for recruitment, allocation, enrolment, analysis and outcome measure

The study will be a randomised, controlled, prospective, parallel-group trial based on the Consolidated Standards of Reporting Trials (CONSORT) statement regarding the randomised trial of non-pharmacological treatments. Children will be randomised to one of two arms – the Small Step Program or treatment as usual. The children in the Small Step Program will be further randomised to start with either hand-use or mobility (Fig. 1). The rationale for the second randomisation is based on the *second* hypothesis.

### The study setting

Astrid Lindgren Children’s Hospital, a tertiary hospital in Stockholm, Sweden.

### Participants

#### Recruitment of subjects

Infants at risk of CP or other neurodevelopmental disorders after neonatal events will be recruited from the regular check-ups that are carried out at 3 months of age within the standard clinical follow-up program at the Astrid Lindgren Children’s Hospital. Children will also be recruited via paediatric neurologists who are examining infants at risk of CP and other neurodevelopmental disorders who are referred to the hospital. These are infants that have typically been exposed to or present with perinatal risk factors such as preterm birth, hypoxia, infections, heart insufficiency, small for gestational age/growth restrictions, hypoxic ischemic encephalopathies (HIE), and morphological brain abnormalities. Infants that exhibit delayed psychomotor development or clinical signs of abnormal neurology will be considered eligible for study participation.

Parents of eligible infants will be informed about the study only after discussions between the medical hospital team and the study coordinator regarding the high-risk status of the child. Families will be given a site-specific information sheet regarding the study, and they will have the opportunity to speak with investigators before consenting to participation in the study. Children of parents who do not wish to participate in the study will continue to receive usual care in the hospital and later on at the habilitation services.

*Inclusion criteria* will be based on a combination of assessments, including the Alberta Motor Infants Scale (using 2SD as the cut-off) and the Hammersmith Infant Neurological Examination (HINE) together with other findings from clinical neurological examinations. Magnetic resonance imaging (MRI) will be used if available to support the risk of development of CP, but only MRI examinations conducted as part of the clinical care will be used. If a child is found to be possibly eligible, the neurological signs indicating risk of CP must also be present at a second examination carried out approximately 3 weeks after the initial assessment before the child is invited to participate in the study. The inclusion age will vary between 4 and 8 months depending on the clarity of signs and time of referral to the hospital. A diagnosis of CP or not will be made by an experienced child neurologist at 2 years of age based on the SCPE criteria (www.scpenetwork.eu), and other neurodevelopmental diagnoses will be based on their specific criteria.

*Exclusion criteria* will be unstable health, uncontrolled epilepsy, progressive disorders, or diagnosis with a specific syndrome. A further exclusion criterion will be children for whom neither parent is fluent in Swedish or English. Satisfactory skills in either language are required for participation in data collection and the coaching and education program.

#### Sample size

The study sample size has been estimated from the pilot project of Morgan et al [19]. Thirty participants (15 per group) has been estimated using motor composite scores of the Peabody Developmental Motor Scale (PDMS-2) with an alpha value of 5 % and power of 80 %, a minimal clinically important difference of 10 %, and a 20 % drop out rate. The Small Step Program has not previously been used, but it has similarities to the method of Morgan et al [19]. However, the design of the Small Step Program requires sub-analysis of growth curves. This requires a higher N, and the planned study sample size is therefore increased to 48 children (24 per group). Interim analysis will be done continuously to control for sample size.

#### Randomisation

The baseline assessments of the children will occur prior to randomization after informed consent is collected. The randomisation is based on a prepared random assignment number list kept by the principal investigator (PI) in a locked room. The children will be allocated to group by block randomisation*.* Stratification will be used to control for gestational age (preterm <37 weeks and term >37 weeks). The infant will be assigned to the next position and added to the list by the PI. If an infant is later excluded for any reason, the infant’s position in the randomisation list will not be replaced by any new infants. Children in the Small Step Program will be further randomised to start with either the mobility or hand use focus.

#### Blinding

The therapists responsible for data collection will not be blinded to group allocation, however, the persons scoring the video-based outcome measures will be blinded to group allocation. Persons involved in data collection will not be involved in the treatment of the children and families. Families will not be blinded to group allocation, but they will be blinded to the study hypotheses.

### Study protocol

The time schedule for enrolment, interventions (including any run-ins and outs), assessments, and participant visits is shown schematically in Fig. 1. Data collection will occur at the start of intervention (baseline), after 14 weeks, post-intervention (approximately 35 weeks), and when the children are 2 years of age (corrected for prematurity) (Fig. 1). Additional data collection in the Small Step group will occur at 7, 21, and 28 weeks after baseline. This means that each step in the program will be evaluated and that hand use and mobility will be trained for two time-periods and communication will be trained for one period during the intervention.

Data collection and neurological examination of the child will take place in the hospital. Parent questionnaires and interviews will take place where it is most convenient for the parents. Project organisation is handled by ACE (the PI), LH, and KL. Data will be stored according to the rules of the Karolinska institutet and only the research group will have access to the final dataset. Reviews of the project process will be planned regularly by the investigation team. We will continuously monitor and reporting adverse event and other unintended effects of trial intervention or trial conduct.

### Small step program

The Small Step Program has three different foci as part of the intervention, but there are general principles that are common for all three areas of the intervention. The rationale for dividing the intervention program into hand use, mobility, and communication is to help the families to optimize the training within each focus area. The general principles of intervention will help parents to see the link between the different foci for training and to better understand the overall idea behind the Small Step Program.

All children will follow the general follow up program at the hospital but during the study period, no other intensive treatment will be provided. If the children are referred to Habilitation Services, the general support will be available but the physiotherapy, occupational therapy, and speech and language therapy will not start until the active study period is finished for the group that is randomized to the Small Step Program.

#### General principle for training

Training will be conducted on a daily basis in the children’s homes with the parents as training providers and with weekly coaching and education from the therapists responsible for the specific focus areas. The individualized goals for training will be adapted to the child’s functional level in all focus areas, and the family will be actively involved in identifying goals for the child. Thus, there will be specific goals for each focus area. The first step in the goal-setting procedure, independent of which focus area is being trained, is to determine the child’s strengths and to identify what self-initiated actions they can perform under optimal conditions. These actions will be the starting point for the training and goal-setting procedure and will be used to determine the expected trajectory of development and progress in all three focus areas. It is important that the goals always reflect the current ability level and that they are not too difficult and not too easy. The goal-setting requires parents to be highly involved, and to ensure this collaboration the therapist will use techniques from motivational interviewing [45]. Intensity and repetition of the goals are important aspects of the program, and the specific activities should be frequently repeated every day during the training periods. The families will be made aware of how much repetition is needed for sustained learning. To ensure that the intended intensity of the program is reached, we will instruct parents to note the amount of time spent practicing each day.

The infant’s self-initiated actions form an important basis in all focus areas. This means that the initiative to move, to use the hands, and to communicate should be the result of the infant’s desire to explore the world as facilitated and stimulated by the training provider. The environment should contain stimuli that capture and drive the child’s actions, and the training provider’s task is to attract and help the child to maintain their attention. To outside observers it should be possible to understand what the child is intended to do, i.e. the training provider has to make sure that the child understands what is expected of them. It is important to support the parent to follow the child’s lead. Thus, the treatment provider should follow and support the child’s actions and not disturb or change the direction of the actions. To stimulate the child’s self-initiated activities, parents are encouraged to use their knowledge of their child’s favourite playtime activities and interests and to use the familiar context and environment at home to support the child’s development. Increased responsiveness to the child’s actions, communicative attempts, and environmental cues will form the key principles of the parent coaching and education that is included in the program. The aim is to help the parents to adapt their responses to the child’s initiative and ability as they develop. Key advice can be: Wait for the child’s initiation, maintain their attention, tempt and encourage the child to act, do not try to force actions, and reinforce and respond positively to the child’s action. The parent should help and guide the child, i.e. be hands-on when necessary and hands-off when not necessary. The home environment for training is chosen because it is important to teach families to use the available possibilities in the child’s daily environment to promote the child’s development. The use and enrichment of the home environment is part of all focus areas. This includes careful toy selection and adaptations of the physical set up, including the positioning and use of conventional baby equipment, to help the parents use their environment in an optimal way.

Although different therapists are responsible for each focus area, the overall aim is to strengthen the parent in the role of being the one who knows what is best for their child. This is also important for parents’ motivation, to organize the training sessions and to actively interact and engage with their child during the training [43]. The “Six-C” model described by Turner and Paris will be used by the therapists when coaching and motivating the parents to be the treatment providers. The six words in the model reflect important aspects of learning from both the child’s and the parents’ perspective. 1. Choice: the parents are actively involved in selecting the goal for where they expect their child to improve. 2. Control: the parents have control of the situation, and the child has control over the activity, at least to some degree. 3. Challenge: the parents are challenged to manage the situation of practicing with their child, and the child is challenged to reach their individual goal. 4. Collaborations: the parents, the child, and the therapists collaborate mutually towards the child’s motor and communicative learning goals. 5. Constructive meaning: the area chosen for the child’s motor and communicative learning is meaningful for both the child and the parents. 6. Consequence: successful motor and communicative learning enables the child to reach more advanced motor and communicative activities. In the coaching process, the families are supported in finding the balance between specified training, everyday expectations, and other family needs. Specific information about the evidence of early intervention will be given and discussed as part of the educational program.

#### Focus areas for training

*Hand use* is based on the assumption that grasping abilities help the infants to explore their environment Development of manual dexterity and different aspects of hand-eye coordination in infants are essential elements of cognitive development. Motor skills and mental processing are developed in conjunction with each other, and both processes are essential for exploring toys. Exciting toys chosen to suit the child’s developmental level and level of hand function will stimulate the child to perform self-initiated actions. Activities on the right level for the child will be important, and for the youngest children and children at the lowest ability level, actions as simple as reaching out to touch their mother’s nose could be meaningful. With the child’s increasing awareness and fine motor development, grasping becomes possible to practice. This provides the opportunity for object exploration, and objects with different properties trigger different hand actions. By using objects with different properties like shape, size, density, and texture, mental processing will be stimulated and will increase the child’s ability to perform repetitions of various actions. With development, children will be stimulated to perform more goal-directed play including simple sequences, like pulling things out of boxes and putting them back in and using both hands in a coordinated manner, for example, to pull things apart. The experience of activities and repetition promotes motor skills as well as higher-order problem-solving skills and leads to more advanced actions. To facilitate hand use, a position that is as upright as possible should be used, and if needed adjustable baby seats and chairs should be used. Most importantly, posture and hand use should not be trained at the same time because insecure posture will influence object exploration. Practice will be centred on the child’s major problems, and this might be motor problems for some children while it might be a lack of interest in object exploration that is the limitation for others. For all children, there is a need to continuously increase variation in object exploration to promote cognitive development.

*Mobility* is based on the assumption that children have an inner drive to move and to explore their environment. The overall objective of the intensive practice of new functional goals of gross motor function will optimize the infant’s possibilities to perform meaningful activities in daily life as they grow older. The training aims to increase postural control and mobility. Training will be tailored in relation to each child with respect to individual strengths and interests. Manual management will be provided to increase the child’s own stability when intending to move and when introducing new functional activities. The manual management is withdrawn gradually as the child demonstrates improved ability to manage and control a sequence of the activity. Less “hands on” assistance will be required, and the children will experience enjoyment with increasing competence in their own body and by learning from trial and error. The manual management is aimed to reduce the degrees of freedom in different joints rather than to facilitate activities as is the case in most previous neurodevelopmental approaches [36]. Once a motor skill is learned, variability of practice is introduced to increase the complexity and generalizability of the skill. To stimulate the child’s gross motor activity and intention to move, parents are encouraged to interact with the child and help them to keep their attention on the tasks being practiced. The environment (different rooms, furniture, and toys) in the child’s home will be arranged to motivate and challenge the child to repeatedly practice towards the expected goals with gradually increasing levels of ability. Parents will also be encouraged to use everyday situations, for example, when the child has to turn around or sit up after changing diapers. Using familiar situations like placing the child in front of a parent’s drawer, which parents often open and pick objects from, will motivate the child to stand up to attempt to reach the objects in the drawer. For children with pronounced motor delays, parents will be encouraged to bring them up into a standing position to promote physical activity and to provide stimulation and experiences of changing positions. Parents will be encouraged to use baby-walkers and other equipment to give the child the opportunity to stand and move around and explore their environment independently.

*Communication training* assumes that all children will have an inner drive to communicate. In the Small Step Program, we will help families to become aware of small signs of intentional communication from their child, which in turn will stimulate communication between parents and their the child. The c*ommunication* framework is mainly based on the Hanen parent program [46]. This implies child-centred communication, i.e. parents are encouraged to actively observe the child’s moment-by-moment focus and to promote the child’s interactions by following the child’s lead by imitating, interpreting, and expanding the child’s vocalisations and non-verbal signals. During the communication intervention, the parents will be guided in how to apply the triad of child-centred, interaction-focused, and language-facilitating communication strategies in their everyday interactions with the child. This approach stresses the importance that the parents try to observe and reinforce all types of communicative attempts the child makes (vocalisations, gazes, gestures, and bodily movements) and to interpret them as meaningful. For a small child, the parent’s face and voice are the most important language markers because they serve as the basis for the development of social cognition. Facial expression together with vocal variation guide the child in understanding which parts of a spoken utterance contains critical information that the child needs in order to direct his/her attention. Here, parents will be educated on the importance of using child-directed speech in their daily communication, thus adapting their communication style to the developmental level of the child. Ideas on play materials and how nursery rhymes and songs might capture and maintain a small child’s attention and support language acquisition are introduced and discussed. Because many of the children in the Small Step Program face physical challenges, Alternative Augmentative Communication techniques might need to be introduced.

#### Usual care

Children in the control group will receive training in accordance with the customary procedure in the hospital follow-up program. This program includes instructions to parents regarding home training. It is not possible to standardise the frequency, intensity, or type of interventions received in the control group because these are based on a combination of resources and the child’s individual needs. However, the families typically meet a physiotherapist for treatment and advice every third week during the first 2–3 months and then about once a month at the hospital. Subsequently, the children will be referred to the Habilitation Services at some time during the first year of life to continue with therapeutic interventions by different professions in accordance with the needs of the individual child. In the Habilitation Service a multidisciplinary team approach is available. The content of therapy in usual care is to support general development and to promote development of functional skills based on the principals of motor learning. Data on the number of appointments for physiotherapy, occupational therapy, and speech and language therapy will be collected after the study is ended. The difference for families included in the control group from those receiving typical customary care will be that they will get feedback on their children’s progress after each examination performed by the researchers at the hospital.

### Outcome measures and procedures

Children and parents in both groups will be assessed at the different endpoints (Fig. 1). The assessments are chosen based on the first aim of the study and to measure general and specific development and describe the characteristics of the brain lesion. Assessments chosen for the second aim evaluate the effects of the program on the parent’s perspective. The *Peabody Developmental Motor Scales, Second Edition (PDMS-2)* will be used as the primary outcome measure.

### Assessments for general development

PDMS-2 is a standard measurement that assesses gross and fine motor skills in young children from birth through age 5 [47]. The PDMS-2 is composed of six subtests that assess related motor abilities that develop early in life. In this study, the Object Manipulation, Grasping, and Visual-Motor Integration subtests will used. The scores on these subtests are presented as percentiles, standard scores, and age equivalents, and the results are used to generate a composite score – the Fine Motor Quotient.

*Bayley Scales of Infant Development (BSID-III)* is a standard measurement to assess the fine and gross motor development, the receptive and expressive language development, and the cognitive development of infants and toddlers ages 0–3 years [48]. This measure consists of a series of developmental play tasks and takes between 45 and 60 min to administer. Raw scores of successfully completed items are converted to scale scores and to composite scores. These scores are used to determine the child’s performance compared with norms taken from typically developing children of the same age in months.

*Hammersmith Infant Neurological Examination (HINE)* is a method to estimate the neurological development of infants aged 2–24 months [49]. It includes three sections: 1) Neurological Exam – tone and movements, 2) Development of Motor Function – head control, sitting, walking, crawling, rolling, and grasping, and 3) State of Behavior – consciousness, social orientation, and emotional state. An optimality score is obtained by calculating the distribution of the frequency of the scores in the normal population. The overall score ranges from a minimum of 0 to a maximum of 78. At 9 or 12 months, scores of 73 and greater are regarded as optimal and below 73 as suboptimal, while at 3 and 6 months healthy term infants have median scores equal to or greater than 67 and 70, respectively.

*Alberta Infant Motor Scale (AIMS)* identifies infants aged 0–18 months who are delayed or deviant in motor development. It is an observational assessment that identifies the gross motor performance of an infant compared to a norm-referenced sample. There are 58 items related to prone, supine, sitting, and standing positions, and the results are reported in a composite score. AIMS has good psychometric properties [50, 51] and has been specially investigated for use with preterm infants [52].

### Assessments for the specific training foci

*Gross Motor Function Measure (GMFM-66)* is an observational, standardized, and criteria-referenced measure that was developed to evaluate changes in gross motor function in children with CP [53, 54]. The items cover gross motor capacity from lying and rolling to walking, running, and jumping. In GMFM-66, the 66 items are organized in increasing difficulty from 0 (low capacity) to 100 (high capacity) along an interval scale (Rasch analysis). Each item is scored on a four-point Likert scale (0–3). It can be used from 5 months of age, and a 5-year-old typically developing child is expected to achieve a score of 100. The GMFM-66 is reported to be valid, reliable, and responsive to changes in gross motor capacity in children with CP [55].

*Hand Assessment for Infants (HAI)* was developed to identify and measure upper limb asymmetry and general manual development from 3 to 12 months in children at risk of developing CP. The test procedure comprises a semi-structured video-recorded play session lasting 10–15 min. A test kit of toys is presented to the infant to encourage and elicit exploration, making a wide range of a unilateral and bilateral hand movements observable. The HAI scale consists of 17 items scored on a three-point grading scale. HAI is still under development, but preliminary Rasch analysis indicates promising results in terms of internal construct validity and unidimensionality.

*Pediatric Evaluation of the Disability Inventory (PEDI)* is a norm and criteria-referenced measure that evaluates functional skills and caregiver assistance in the domains of self-care, mobility, and social function [56]. The child is assessed through structured interviews with the parents. The summary scores can be converted to normative standard scores and scaled scores, and normative score are available for children aged 6 months to 7.5 years. The scaled scores range on a continuum from 0 to 100. PEDI is available in a Swedish version (Nordmark [57].

*Parent-child Early Relational Assessment (PC-ERA)* measures parent’s and the child’s affect and behavioural characteristics. Ratings are based on observations of videotaped parent-child interactions in three types of situations – free play, feeding, and a structured task [58, 59]. The purpose of the method is to capture the child’s experience of the parent, the parent’s experience of the child, the affective and behavioural characteristics that both bring into the interaction, and the quality or tone of the relationship. The reliability and validity of the method have been addressed in both high-risk and normative populations with positive outcomes.

*Swedish Early Communicative Development Inventory (SECDI)* is a parent questionnaire and is the Swedish version of The MacArthur Communicative Development Inventories (CDI). CDI is an internationally recognized parent reporting instrument for assessing early language development in children. The reliability and validity of SECDI has been investigated thoroughly and has been found to be satisfactory [60, 61]. The instrument consists of two separate inventories: Words and Gestures (CDI/WG) for children 8 to 16 months and Words and Sentences (CDI/WS) for children 16 to 30 months.

### Examinations used to describe brain pathology

*Structural magnetic resonance imaging (MRI)* will be used to investigate the third hypothesis of this study. Brain lesion characteristics (i.e. type, location and extent) will be described using MRI acquired for clinical purposes. The age at imaging will thus vary, but images collected when the child is older than 6 months will primarily be used. All MRIs will be visually assessed specifically for this study by experienced neuroradiologists unaware of the infants’ clinical diagnosis and functional outcome. The analytical protocol was developed in our group and has been applied in a previous study [62] using the primary patterns of abnormality defined and described by Ashwal [63].

### Assessments evaluating the parent’s perspective

*Responsive Augmentative and Alternative Communication Style Scale (RAACS) Version 3* was developed for the purpose of assessing parents’ communicative styles with children who have communication difficulties [64]. The assessment is based on a video of free communication between parents and their children lasting a maximum of 10 min. It is a criteria-based assessment in which the films are coded on seven domains with a three-point grading scale. The results from the RAACS Version 3 can be used for planning and interventions as well as an outcome measure.

*Swedish Parenthood Stress Questionnaire (SPSQ)* measures perceived parental stress and is a revised version of the parent domain of the Parenting Stress Index. The instrument contains 34 items within the five sub-areas of *Incompetence regarding parenthood*, *Role restriction*, *Social isolation*, *Spouse relationship problems*, and *Health problems* [65]. The response options range from “Strongly agree” to “Strongly disagree” on a Likert scale of 1 to 5, with a total score of 170. Higher scores indicate higher stress. The SPSQ has been found to be a valid and reliable instrument [66].

*The Hospital Anxiety and Depression Scale (HADS*) is a self-assessment scale developed to detect states of depression, anxiety, and emotional distress among patients who are being treated for a variety of clinical problems [67]. The HADS is a fourteen-item scale that generates ordinal data, and higher scores indicate more distress. Prior to completing the scale, patients are asked to *“fill it completely in order to reflect how they have been feeling during the past week*”. HADS is translated into Swedish.

*The Working Model of the Child Interview (WMCI)* is a semi-structured, open-ended interview designed to assess parent’s representations of their infant/child and their relationship with their infant/child [68]. The interview is videotaped and takes approximately 1 h. The WMCI is coded and summarized into classification types reflecting the parents’ overall state of mind with respect to their relationship with their infant/child. The WMCI has been found to be both a reliable and valid approach to scoring representational aspects of parent-child relationships.

## Statistical methods

Descriptive data for each outcome measure (primary and secondary) will be summarized and presented for both treatment groups (Small Step or treatment as usual). Changes over time and developmental trajectories will be investigated in both groups for each outcome measure with the use of mixed models, repeated-measures ANOVA, or corresponding non-parametric options depending on the data level and the distribution of data and in accordance with guidelines for RCTs. Post hoc analysis with correction for multiple comparisons will be applied when suitable. Group comparisons on all outcome measures will be made at 14 weeks and approximately 35 weeks after the start of the intervention and when the participants are 2 years old. All analyses will be conducted using IBM SPSS Statistics 22, and a significance level of *p* < 0.05 will be used.

## Discussion

By developing the Small Step Program and using it with children at risk for neurodevelopmental disorders, we aim to influence the affected developmental trajectories in a positive way so that they approach what is expected in typically developing children. We do not think the program will prevent children from developing CP, but the aim is to get the children to function on a higher level than if not treated by the program. If the program is effective, the new knowledge generated will have an important impact for planning both short and long-term treatment and services.

## Abbreviations

AIMS: Alberta infant motor scale
BSID-III: Bayley scales of infant development
CIMT: Constraint-induced movement therapy
CONSORT: Consolidated standards of reporting trials
COPCA: COPing with and CAring for Infants with special needs
CP: Cerebral palsy
GMFM: Gross motor function measure
HADS: The Hospital anxiety and depression scale
HAI: Hand assessment for infants
HINE: Hammersmith infant neurological examination
MRI: Structural magnetic resonance imaging
PC-ERA: Parent-child early relational assessment
PDMS-2: Peabody developmental motor scales, second edition
PEDI: Pediatric evaluation of the disability inventory
RAACS: Responsive augmentative and alternative communication style scale
SCPE: Surveillance cerebral palsy evaluation
SECDI: Swedish early communicative development inventory
SPSQ: Swedish parenthood stress questionnaire
WMCI: The working model of the child interview
ZPD: Zone of proximal development

## References

[1] Christine C, Dolk H, Platt MJ, Colver A, Prasauskiene A, Krageloh-Mann I, Group SC (2007). Recommendations from the SCPE collaborative group for defining and classifying cerebral palsy. Dev Med Child Neurol Suppl.

[2] Hadders-Algra M (2011). Challenges and limitations in early intervention. Dev Med Child Neurol.

[3] McIntyre S, Morgan C, Walker K, Novak I (2011). Cerebral palsy--don’t delay. Dev Disabil Res Rev.

[4] McIntyre S, Taitz D, Keogh J, Goldsmith S, Badawi N, Blair E (2013). A systematic review of risk factors for cerebral palsy in children born at term in developed countries. Dev Med Child Neurol.

[5] Keogh JM, Badawi N (2006). The origins of cerebral palsy. Curr Opin Neurol.

[6] Bax M, Tydeman C, Flodmark O (2006). Clinical and MRI correlates of cerebral palsy: the European Cerebral Palsy Study. JAMA.

[7] Skiold B, Eriksson C, Eliasson AC, Aden U, Vollmer B (2013). General movements and magnetic resonance imaging in the prediction of neuromotor outcome in children born extremely preterm. Early Hum Dev.

[8] Spittle AJ, Boyd RN, Inder TE, Doyle LW (2009). Predicting motor development in very preterm infants at 12 months’ corrected age: the role of qualitative magnetic resonance imaging and general movements assessments. Pediatrics.

[9] Krageloh-Mann I, Horber V (2007). The role of magnetic resonance imaging in furthering understanding of the pathogenesis of cerebral palsy. Dev Med Child Neurol.

[10] Reid SM, Dagia CD, Ditchfield MR, Carlin JB, Meehan EM, Reddihough DS (2014). An Australian population study of factors associated with MRI patterns in cerebral palsy. Dev Med Child Neurol.

[11] Wiklund LM, Uvebrant P (1991). Hemiplegic cerebral palsy: correlation between CT morphology and clinical findings. Dev Med Child Neurol.

[12] Friel KM, Chakrabarty S, Martin JH (2013). Pathophysiological mechanisms of impaired limb use and repair strategies for motor systems after unilateral injury of the developing brain. Dev Med Child Neurol.

[13] Martin JH, Chakrabarty S, Friel KM (2011). Harnessing activity-dependent plasticity to repair the damaged corticospinal tract in an animal model of cerebral palsy. Dev Med Child Neurol.

[14] Riethmuller AM, Jones R, Okely AD (2009). Efficacy of interventions to improve motor development in young children: a systematic review. Pediatrics.

[15] Blauw-Hospers CH, Hadders-Algra M (2005). A systematic review of the effects of early intervention on motor development. Dev Med Child Neurol.

[16] Orton J, Spittle A, Doyle L, Anderson P, Boyd R (2009). Do early intervention programmes improve cognitive and motor outcomes for preterm infants after discharge? A systematic review. Dev Med Child Neurol.

[17] Novak I, McIntyre S, Morgan C, Campbell L, Dark L, Morton N, Stumbles E, Wilson SA, Goldsmith S (2013). A systematic review of interventions for children with cerebral palsy: state of the evidence. Dev Med Child Neurol.

[18] Morgan C, Novak I, Badawi N (2013). Enriched environments and motor outcomes in cerebral palsy: systematic review and meta-analysis. Pediatrics.

[19] Morgan C, Novak I, Dale RC, Badawi N (2015). Optimising motor learning in infants at high risk of cerebral palsy: a pilot study. BMC Pediatr.

[20] Hielkema T, Blauw-Hospers CH, Dirks T, Drijver-Messelink M, Bos AF, Hadders-Algra M (2011). Does physiotherapeutic intervention affect motor outcome in high-risk infants? An approach combining a randomized controlled trial and process evaluation. Dev Med Child Neurol.

[21] Spittle A, Orton J, Anderson P, Boyd R, Doyle LW (2012). Early developmental intervention programmes post-hospital discharge to prevent motor and cognitive impairments in preterm infants. Cochrane Database Syst Rev.

[22] Milgrom J, Newnham C, Martin PR, Anderson PJ, Doyle LW, Hunt RW, Achenbach TM, Ferretti C, Holt CJ, Inder TE (2013). Early communication in preterm infants following intervention in the NICU. Early Hum Dev.

[23] Nordstrand L, Holmefur M, Kits A, Eliasson AC (2015). Improvements in bimanual hand function after baby-CIMT in two-year old children with unilateral cerebral palsy: A retrospective study. Res Dev Disabil.

[24] Whittingham K, Wee D, Boyd R (2011). Systematic review of the efficacy of parenting interventions for children with cerebral palsy. Child Care Health Dev.

[25] Lowing K, Bexelius A, Brogren CE (2009). Activity focused and goal directed therapy for children with cerebral palsy--do goals make a difference?. Disabil Rehabil.

[26] Vroland-Nordstrand K, Eliasson AC, Jacobsson H, Johansson U, Krumlinde-Sundholm L. Can children identify and achieve goals for intervention? A randomized trial comparing two goal-setting approaches. Dev Med Child Neurol. 2015;58(6):589–96. 10.1111/dmcn.12925 26374194

[27] Locke EA, Latham GP (2002). Building a practically useful theory of goal setting and task motivation. A 35-year odyssey. Am Psychol.

[28] Thelen E, Smith LB (1996). A dynamic systems approach to development of cognition and action.

[29] Smith RA, Wrisberg CA (2001). Motor learning and performance. A problem-based learning approach.

[30] Vygotski LS, Kozulin A (1986). Thought and language.

[31] Adolph KE, Vereijken B, Shrout PE (2003). What changes in infant walking and why. Child Dev.

[32] Baldwin P, King G, Evans J, McDougall S, Tucker MA, Servais M (2013). Solution-focused coaching in pediatric rehabilitation: an integrated model for practice. Phys Occup Ther Pediatr.

[33] von Hofsten C, Lindhagen K (1979). Observations on the development of reaching for moving objects. J Exp Child Psychol.

[34] Fagard J, Jacquet A (1989). Onset of bimanual coordination and symmetry versus asymmetry of movement. Infant Behav Dev.

[35] Bennet GC, Harrold AJ (1976). Prognosis and early management of birth injuries to the brachial plexus. BMJ.

[36] Shumway-Cook A, Wolpaw JR (2007). Motor control:translating research into clinincal practice.

[37] Dunst C, Lowe L (1986). From reflex to symbol: describing, explaining, and fostering communicative competence. Augment Altern Commun.

[38] Mundy P, Newell L (2007). Attention, joint attention, and social cognition. Curr Dir Psychol Sci.

[39] Hobson RP, Eilan N, Hoerl C, McCormack T, Rossler J (2005). What puts the jointness into joint attention. Joint attention: communication and other minds.

[40] Graham F, Rodger S, Ziviani J (2010). Enabling occupational performance of children through coaching parents: three case reports. Phys Occup Ther Pediatr.

[41] Dirks T, Hadders-Algra M (2011). The role of the family in intervention of infants at high risk of cerebral palsy: a systematic analysis. Dev Med Child Neurol.

[42] Blauw-Hospers CH, Dirks T, Hulshof LJ, Bos AF, Hadders-Algra M (2011). Pediatric physical therapy in infancy: from nightmare to dream? A two-arm randomized trial. Phys Ther.

[43] Turner J, Paris SG (1995). How literacy tasks influence children’s motivation for literacy. Read Teach.

[44] Novak I (2014). Evidence to practice commentary new evidence in coaching interventions. Phys Occup Ther Pediatr.

[45] Miller W, Rollnick S (2002). Motivational interviewing: preparing people for change.

[46] Pepper J, Weitzman E (2004). It takes two to talk. A practical guide for parents of children with language delays.

[47] Folio MR, Fewell RR. Peabody developmental motor scales, PDMS-2, vol. 2nd. Austin: Pro ED; 2000.

[48] Bayley N (2006). Bayley scale of infant and toddler development.

[49] Dubowitz LM, Dubowitz V (1981). Neurological assessment of the preterm and fullterm newborn infant. Clinics in Developmental Medicine No79.

[50] Darrah J, Piper M, Watt MJ (1998). Assessment of gross motor skills of at-risk infants: predictive validity of the Alberta Infant Motor Scale. Dev Med Child Neurol.

[51] Darrah J, Redfern L, Maguire TO, Beaulne AP, Watt J (1998). Intra-individual stability of rate of gross motor development in full-term infants. Early Hum Dev.

[52] Pin TW, de Valle K, Eldridge B, Galea MP (2010). Clinimetric properties of the Alberta infant motor scale in infants born preterm. Pediatr Phys Ther.

[53] Russel DJ, Avery LM, Rosenbaum PL, Raina PS, Walter SD, Palisano RJ (2000). Improved scaling of the gross motor function measure for children with cerebral palsy: evidence of reliablility and validity. Phys Ther.

[54] Russell DJ, Rosenbaum P, Avery L, Lane M (2002). Gross Motor Function Measure (GMFM-66 & GMFM 88).

[55] Vos-Vromans DC, Ketelaar M, Gorter JW (2005). Responsiveness of evaluative measures for children with cerebral palsy: the gross motor function measure and the pediatric evaluation of disability inventory. Disabil Rehabil.

[56] Haley SM, Coster WL, Ludlow LH, Haltiwanger JT, Andrellos PJ (1992). Pediatric evaluation of disability inventory (PEDI). Development, standardization and administration manual.

[57] Nordmark E, Orban K, Hagglund G, Jarnlo GB (1999). The American Paediatric Evaluation of Disability Inventory (PEDI). Applicability of PEDI in Sweden for children aged 2.0-6.9 years. Scand J Rehabil Med.

[58] Clark RA (1985). The parent-child early relational assessment: Instrument and manual.

[59] Clark R (1999). The parent-child early relational assessment: a factorial validity study. Educ Psychol Meas.

[60] Berglund E, Eriksson M (2000). Reliability and content validity of a new instrument for assessment of communicative skills and language abilities in young Swedish children. Logoped Phoniatr Vocol.

[61] Berglund E, Eriksson M (2000). Communicative development in Swedish children 16-28 months old: the Swedish early communicative development inventory--words and sentences. Scand J Psychol.

[62] Holmstrom L, Vollmer B, Tedroff K, Islam M, Persson JK, Kits A, Forssberg H, Eliasson AC (2010). Hand function in relation to brain lesions and corticomotor-projection pattern in children with unilateral cerebral palsy. Dev Med Child Neurol.

[63] Ashwal S, Russman BS, Blasco PA, Miller G, Sandler A, Shevell M, Stevenson R (2004). Practice parameter: diagnostic assessment of the child with cerebral palsy: report of the Quality Standards Subcommittee of the American Academy of Neurology and the Practice Committee of the Child Neurology Society. Neurology.

[64] Broberger U, Forssberg H, Lagercrantz H, Katz-Salamon M. Morbidity and mortality in 262 infants with birth weight below 1500 g born in Stockholm County 1988-1992. Acta Ophthalmol Suppl. 1993;(210):34-36. 10.1111/j.1755-3768.1993.tb04148.x 8329950

[65] Ostberg M, Hagekull B, Wettergren S (1997). A measure of parental stress in mothers with small children: dimensionality, stability and validity. Scand J Psychol.

[66] Ostberg M (1998). Parental stress, psychosocial problems and responsiveness in help-seeking parents with small (2-45 months old) children. Acta Paediatr.

[67] Zigmond AS, Snaith RP (1983). The hospital anxiety and depression scale. Acta Psychiatr Scand.

[68] Zeanah CH, Benoit D, Barton M, Hirshberg L (1996). Working model of child interview coding manual.

